# Drug Penetration into the Central Nervous System: Pharmacokinetic Concepts and In Vitro Model Systems

**DOI:** 10.3390/pharmaceutics13101542

**Published:** 2021-09-23

**Authors:** Felix Neumaier, Boris D. Zlatopolskiy, Bernd Neumaier

**Affiliations:** 1 Institute of Radiochemistry and Experimental Molecular Imaging, Faculty of Medicine and University Hospital Cologne, University of Cologne, Kerpener Str. 62, 50937 Cologne, Germany; boris.zlatopolskiy@uk-koeln.de (B.D.Z.); b.neumaier@fz-juelich.de (B.N.); 2 Forschungszentrum Jülich GmbH, Institute of Neuroscience and Medicine, Nuclear Chemistry (INM-5), Wilhelm-Johnen-Str., 52428 Jülich, Germany

**Keywords:** BBB permeability, parallel artificial membrane permeability assay (PAMPA), immobilized artificial membrane (IAM) chromatography, static BBB model, dynamic BBB model, microfluidic BBB model, positron emission tomography (PET), neurotracer, monolayer, co-culture model

## Abstract

Delivery of most drugs into the central nervous system (CNS) is restricted by the blood–brain barrier (BBB), which remains a significant bottleneck for development of novel CNS-targeted therapeutics or molecular tracers for neuroimaging. Consistent failure to reliably predict drug efficiency based on single measures for the rate or extent of brain penetration has led to the emergence of a more holistic framework that integrates data from various in vivo, in situ and in vitro assays to obtain a comprehensive description of drug delivery to and distribution within the brain. Coupled with ongoing development of suitable in vitro BBB models, this integrated approach promises to reduce the incidence of costly late-stage failures in CNS drug development, and could help to overcome some of the technical, economic and ethical issues associated with in vivo studies in animal models. Here, we provide an overview of BBB structure and function in vivo, and a summary of the pharmacokinetic parameters that can be used to determine and predict the rate and extent of drug penetration into the brain. We also review different in vitro models with regard to their inherent shortcomings and potential usefulness for development of fast-acting drugs or neurotracers labeled with short-lived radionuclides. In this regard, a special focus has been set on those systems that are sufficiently well established to be used in laboratories without significant bioengineering expertise.

## 1. Introduction

To maintain normal brain homeostasis, the exchange of many substances between blood and the central nervous system (CNS) is restricted by two dedicated biological barriers, the blood–brain barrier (BBB) and the blood–cerebrospinal fluid (CSF) barrier (BCSFB). The BBB is primarily formed by the endothelium that makes up the wall of all brain capillaries, while the BCSFB is located at the level of the choroid plexus and is formed by the tight epithelium lining the ventricles. With an estimated surface area of 10–20 m^2^ or 100–240 cm^2^/g brain in humans [1,2,3,4,5,6], the BBB is almost 5000-fold larger than the BCSFB, which makes it the primary interface for exchange of compounds between circulation and CNS [7]. Although it is crucial for import of nutrients and export of metabolites from the CNS, the BBB also restricts delivery of most drugs to the brain [2] and represents a significant bottleneck in the development of novel CNS-targeted therapeutics or neurotracers for imaging techniques like positron emission tomography (PET). In vivo studies in animal models can be used to assess drug penetration across the BBB, but they are associated with a number of technical, economic and ethical issues, especially when it comes to high-throughput evaluation of large compound libraries. As a consequence, a number of in vitro approaches have been introduced to complement or replace in vivo studies for early estimation of brain penetration by candidate drugs. With regard to in vitro models of the BBB, a number of excellent reviews have addressed the history, current state and future directions of the field [8,9,10,11,12,13], but there is still uncertainty concerning their actual value for the drug development process. For example, while estimates for the rate of drug transport across the BBB derived from in vitro models have long been used to identify promising candidates for development of CNS-targeted therapeutics, the rate of BBB penetration per se is now recognized as a poor predictor for the effectiveness of drugs that are dosed continuously [14,15]. On the other hand, in vitro approaches for estimating the rate of BBB penetration can be very useful if the aim is to develop fast-acting drugs like anticonvulsants or PET tracers labeled with short-lived radionuclides. Thus, even successful CNS-targeted therapeutics with high brain penetration may be useless for PET imaging if their transfer across the BBB is so slow that equilibration takes several hours or days. Further complexity arises from the fact that the actual time required for equilibration of a drug between blood and brain is not exclusively determined by the rate of BBB penetration, and may also depend on factors such as non-specific drug binding to brain tissue or the rate of drug delivery by cerebral blood flow (CBF). With this in mind, the aim of the present article is two-fold: to review the pharmacokinetic parameters that influence the rate or extent of drug penetration into the brain, and to provide an overview of different models that can be used to study BBB penetration in vitro. To this end, Section 2 will briefly summarize current knowledge regarding BBB structure and function in vivo, while Section 3 will describe how drug penetration into the brain can be quantified or estimated based on a combination of in vivo, in situ and in vitro data. Finally, Section 4 will address the most common in vitro models of the BBB in terms of their advantages and limitations. A list of all abbreviations used in the text as well as a summary of the most important equations and parameters are provided in the Supplementary Materials.

## 2. Structure and Function of the BBB

**Figure 1:**
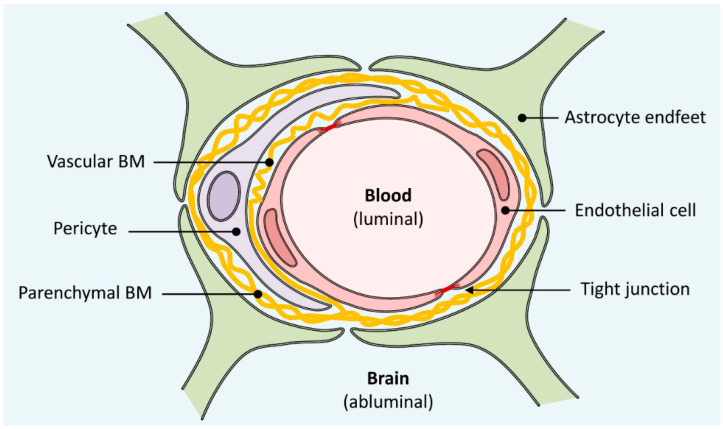
Anatomical structure of the blood–brain barrier (BBB). The wall of all brain capillaries is formed by a thin monolayer of specialized brain microvascular endothelial cells joined together by tight junctions, which act as a physical, transport and metabolic barrier. They are surrounded by a vascular basement membrane (BM), pericytes, a parenchymal BM and astrocyte endfeet, all of which directly or indirectly contribute to the barrier function of the BBB.

**Figure 2:**
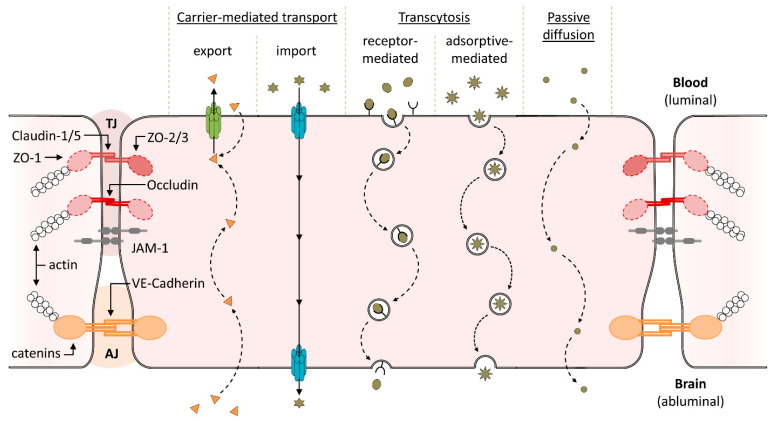
Molecular structure and function of the brain microvascular endothelium. Structural support is provided by adherens junctions (AJ), which are formed by interaction of vascular endothelial (VE)-cadherin from adjacent cells and anchored to the actin cytoskeleton through catenins. The physical barrier function results from tight junctions (TJ), which restrict paracellular permeability and are formed by interaction of claudins, occludin and junctional adhesion molecules like JAM-1 from adjacent cells. Both claudins and occludin are anchored to the actin cytoskeleton through membrane-associated zonula occludens (ZO) proteins. The transport barrier function results from export of lipophilic xenobiotics and drugs (orange triangles) by efflux transporters (indicated in green) present in the luminal (i.e., blood-sided) membrane. Transfer of nutrients and other compounds into the brain depends on their physicochemical and/or biological properties and can occur through carrier-mediated import, receptor- or adsorptive-mediated transcytosis or passive diffusion. Finally, intracellular metabolic enzymes (not shown) can metabolize compounds on their way into the brain, conferring the endothelium with an additional, metabolic barrier function.

The anatomical substrate of the BBB is a thin monolayer of brain microvascular endothelial cells (BMECs) which form the wall of all brain capillaries and make intimate contact with other cells of the neurovascular unit, most notably pericytes and astrocytes (Figure 1). Pericytes are discontinuously distributed along the capillary walls and surround the BMECs with an estimated coverage of ~30%, while the endfeet of astrocytes almost completely envelope the abluminal side of the capillaries, with an estimated coverage of 99% [16]. Structural support and contact points for cell anchoring are provided by an inner (vascular) and an outer (parenchymal) basement membrane (BM), which also serve as another barrier for molecules and cells entering the CNS [17,18]. Both BMs are mainly composed of extracellular matrix (ECM) proteins (collagen type IV, laminins, nidogen and perlecan) that are either secreted by nearby BMECs and pericytes (vascular BM) or by nearby astrocytes (parenchymal BM), respectively [17]. In contrast to most peripheral endothelial cells, BMECs lack fenestrations and show very low levels of non-specific pinocytosis. In addition, they are closely joined together by adherens junctions (AJs), which provide structural support; and tight junctions (TJs), which seal the intercellular cleft and restrict paracellular permeability of the endothelium [6,19,20]. TJs are dynamic complexes formed by interaction of integral transmembrane proteins (e.g., claudins, occludin, junctional adhesion molecules) from adjacent BMECs, and are anchored to the actin cytoskeleton by membrane-associated cytoplasmic scaffolding proteins (mainly zonula occludens proteins) (Figure 2) [6,21,22,23]. This results in a physical barrier with high transendothelial electrical resistance (TEER) of >1000 Ω × cm^2^ and very low permeability to small polar solutes like sucrose, as determined by in vivo measurements in anesthetized or conscious rats [24,25,26]. The endothelium also acts as a transport and metabolic barrier, since BMECs express various transporters and metabolic enzymes that can transform, import or export compounds from the brain [6,7,27,28,29]. Most notably, multidrug resistance proteins (MRPs) like P-glycoprotein (PGP/MDR1) or ATP-binding cassette (ABC) transporters present on the luminal (i.e., blood-sided) membrane are responsible for efflux of many lipophilic xenobiotics and drugs from the CNS (Figure 2) [30,31]. In addition, BMECs are equipped with several pathways for the import of nutrients and other compounds into the CNS [6]. This includes transport of glucose and amino acids by specific solute carriers in luminal and abluminal (i.e., CNS-sided) membrane, of macromolecules like insulin and transferrin or albumin by receptor- or adsorptive-mediated transcytosis [6,32,33], and of small lipophilic molecules or gases like O_2_ by passive diffusion (Figure 2) [6]. The latter is usually regarded as the most important pathway for penetration of small molecule-based drugs into the CNS, although there is some evidence that the role of specific transporters may be more significant than previously thought [34]. The exact tightness and transporter activity of the BBB are highly dynamic and vary in response to local environmental and systemic influences [35]. For example, interactions between TJs and AJs are thought to modify the barrier properties through changes in paracellular permeability (via signaling pathways from the cytoplasm to TJs) and BMEC transporter expression (via signaling pathways from TJs to the cytoplasm) [21]. In addition, both pericytes and astrocytes have been shown to regulate the phenotype of the endothelium through paracrine mechanisms and possibly direct contact interactions [5,36,37]. Thus, astrocytes secrete a number of signaling molecules like glia cell-derived neurotropic factor (GDNF), basic fibroblast growth factor (bFGF) or transforming growth factor β (TGF-β), which have all been shown or are thought to regulate the BBB by altering BMEC transporter expression [38,39,40,41] or the paracellular tightness of the endothelium [42]. Soluble growth factors can also bind to ECM proteins, which participate in their distribution, activation and presentation to cells or directly transduce signals into the cytoplasm [43,44]. Another factor that alters the barrier function and reduces permeability of the BBB through increased expression of transporters and TJs in BMECs is the shear stress generated by CBF [45]. As described in Section 4, all of these factors have important implications for the generation of reliable in vitro models of the BBB.

## 3. In Vivo and In Vitro Descriptors of Brain Penetration

**Figure 3:**
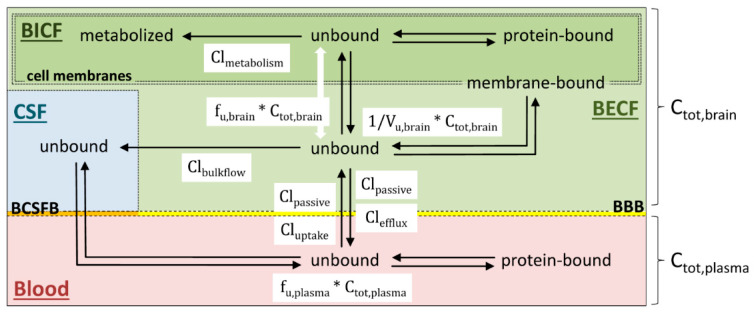
Overview of the equilibria involved in CNS penetration by drugs and their intra-brain distribution. The total concentration of a drug in plasma (*C_tot,plasma_*) is the sum of protein-bound and unbound drug species, of which only the unbound fraction (*f_u,plasma_*) can penetrate the blood brain barrier (BBB) or blood cerebrospinal fluid barrier (BCSFB), respectively. Drug transfer from blood to brain extracellular fluid (BECF) is usually driven by diffusional clearance (*Cl_passive_*) and active uptake transporter clearance (*Cl_uptake_*) of the drug across the BBB. Once it has entered the brain, the unbound drug (middle) may bind to its target (if the target is extracellular), and/or nonspecifically bind to brain tissue (middle right) and/or be cleared from BECF through various pathways. In particular, the unbound drug may be removed back to plasma through diffusional clearance (*Cl_passive_*) and/or active efflux (*Cl_efflux_*) across the BBB (bottom), it may be cleared due to bulk flow of BECF (*Cl_bulkflow_*) into CSF (left), and/or it may enter the brain intracellular fluid (BICF) due to uptake into cells (top). Likewise, within BICF, the unbound drug (top middle) may bind to its target (if the target is intracellular), and/or it may become bound to intracellular proteins (top right), and/or it may be cleared by metabolic enzymes (*Cl_metabolism_*) in the cells (top left). Note that drug metabolism may also take place at the BBB or in BECF, which has been omitted for clarity. Because the unbound drug fraction (*f_u,brain_*) is determined in homogenized tissue, it lumps together the unbound drug fractions in BECF and BICF. In contrast, the unbound volume of drug distribution (*V_u,brain_*) is determined by in vivo microdialysis or in brain slices, so that it provides a measure for the unbound drug fraction in BECF.

Delivery of a given drug from blood to brain can be described in terms of its rate (i.e., the speed at which the drug enters the brain) and its extent (i.e., the amount of drug that reaches the brain) [14], both of which differ in their determinants and implications for drug development. Because they have often been used or interpreted incorrectly [14,15], a brief recapitulation of how rate and extent of brain entry can be measured and related to each other seems warranted. A simplified scheme of the relevant brain compartments and the equilibria between them, blood and CSF is provided in Figure 3.

### 3.1. Extent of Brain Penetration

The extent of brain penetration by a given drug has traditionally been determined by measuring the concentration of drug in brain tissue homogenate and plasma samples obtained at different time points after administration in rodents. The concentration ratio of drug in brain and blood can then be described by the partition coefficient *K_p,brain_*

(1) $$K_{p,brain}=\frac{C_{tot,brain}}{C_{tot,plasma}}$$

where *C_tot_*_,*brain*_ and *C_tot_*_,*plasma*_ are the total drug concentrations at steady-state (after continuous infusion) or areas under the concentration-time curves (after bolus injection) in brain tissue homogenate and plasma, respectively. Provided that the drug can be radiolabeled without altering its structure (i.e., ^11^C- or ^18^F-labeled isotopologes of the non-radioactive compounds), *K_p_*_,*brain*_ can also be determined from PET measurements, either by comparing steady-state tracer concentrations in brain and plasma [46], or by compartmental modeling [47] or by model-independent graphical analysis [48,49]. Due to differences between PET and standard pharmacokinetic nomenclature, the partition coefficient determined in PET measurements is usually referred to as volume of distribution (V_T_) [50,51,52], which is equivalent to *K_p_*_,*brain*_ and should not be confused with the unbound volume of distribution of a drug determined in vitro in brain slices (*V_u_*_,*brain*_), which will be described later in this subsection.

*K_p_*_,*brain*_ has been widely used as a basis for in silico prediction of brain drug exposure, usually in terms of its logarithm (*logBB*) [53]. However, since it is derived from the measurement of total rather than unbound drug concentrations, *K_p_*_,*brain*_ lumps together brain partitioning, non-specific binding of the drug to brain tissue, and protein binding in plasma [14,15]. In addition, *K_p_*_,*brain*_ or *logBB* values can differ between CNS-active drugs by a factor of up to at least 2000-fold [14], which limits their value for identification of promising candidates. Because only the unbound drug can cross the BBB and exert its physiological action, a much more useful measure for BBB partitioning and brain penetration can be obtained by correcting for drug binding to proteins in plasma and drug distribution in the brain. This is achieved by calculation of the unbound partition coefficient *K_p_*_,*uu*,*brain*_ according to

(2) $$K_{p,uu,brain}=\frac{K_{p,brain}}{f_{u,plasma}\times V_{u,brain}}$$

where *f_u_*_,*plasma*_ is the unbound drug fraction in plasma and *V_u_*_,*brain*_ (in mL/g brain) is the unbound volume of distribution of the drug in brain. *V_u_*_,*brain*_ describes the relationship between total drug concentration in brain and (unbound) drug concentration in brain extracellular fluid (BECF) [14,54,55]. It can be determined from drug concentrations in brain measured by in vivo microdialysis after administration in rodents according to

(3) $$V_{u,brain}=\frac{A_{tot,brain+blood}-V_{blood}\times C_{tot,plasma}}{C_{BECF}}$$

where *C_BECF_* is the (by definition unbound) drug concentration in cerebral microdialysate, *A_tot_*_,*brain*+*blood*_ is the total amount of drug per g of brain tissue (including blood) determined after the in vivo experiment, and *V_blood_* is the volume of blood in the brain tissue [14,56].

However, due to several technical and methodological issues [57,58], the in vivo approach is rarely used in the drug development setting, and *V_u_*_,*brain*_ is instead usually determined by in vitro uptake studies in brain slices. These studies involve incubation of a brain slice in buffer containing the drug and calculation of *V_u_*_,*brain*_ according to

(4) $$V_{u,brain}=\frac{A_{tot,slice}}{C_{buffer}}$$

where *A_tot_*_,*slice*_ is the total amount of drug per g of brain slice tissue measured after the incubation, and *C_buffer_* is the (unbound) drug concentration in the incubation buffer [14,55,59,60]. Based on the total volumes of BECF (~0.2 mL/g brain) and brain intracellular fluid (BICF~0.6 mL/g brain), a *V_u_*_,*brain*_ in the order of 0.2 mL/g (~BECF) indicates that most of the drug is unbound and present in BECF, while a value in the order of 0.8 mL/g (~BECF + BICF) indicates that the drug distributes equally between BECF and BICF [61]. Values above 0.8 mL/g point to strong nonspecific binding of the drug to brain tissue and/or active transport into brain cells with subsequent lysosomal trapping or sequestration into other cell organelles [15,61].

A widely used alternative to *V_u_*_,*brain*_ is the unbound drug fraction (*f_u_*_,*brain*_), which can be determined by equilibrium dialysis with brain tissue homogenate [60,62,63]. This involves dilution of the brain homogenate in a buffer solution and equilibration with the drug across a dialysis membrane, after which *f_u_*_,*brain*_ can be calculated according to

(5) $$f_{u,brain}=\frac{1/D}{\left[\left(\frac{1}{f_{u,dh}}\right)-1\right]+\;\left(1/D\right)}$$

where *f_u_*_,*dh*_ is the fraction of unbound drug in the diluted tissue homogenate and *D* is the dilution factor. The resulting value of *f_u_*_,*brain*_ can then be used to calculate *K_p_*_,*uu*,*brain*_ according to the following equation

(6) $$K_{p,uu,brain}=K_{p,brain}\times \frac{f_{u,brain}}{f_{u,plasma}}$$

This approach has the advantage that *f_u_*_,*brain*_ can be determined in a high-throughput format and with the same equipment as that used for the determination of *f_u_*_,*plasma*_ [64]. In addition, because brain composition is highly conserved across species, *f_u_*_,*brain*_ is essentially species-independent [51,65]. On the other hand, the in vivo interpretation of *K_p_*_,*uu*,*brain*_ with this method is complicated by the fact that homogenizing brain tissue destroys most intratissue compartments, so that *f_u_*_,*brain*_ does not take into account intracellular drug distribution and mainly measures nonspecific binding [14]. As such, *f_u,brain_* only corresponds to 1/*V_u_*_,*brain*_ if a drug shows roughly equal distribution between BECF and BICF. One approach to overcome this limitation is based on the observation that the most pronounced differences between *f_u_*_,*brain*_ and 1/*V_u_*_,*brain*_ occur for basic compounds that exhibit lysosomal trapping [66,67]. Provided that the pK_a_ values of the drug and the pH values in the different compartments are known, the Henderson–Hasselbalch equation can be used to correct for lysosomal trapping, in which case the *f_u_*_,*brain*_ for a number of basic compounds has been shown to be approximately equal to 1/*V_u_*_,*brain*_ within a two-fold range [66,67].

### 3.2. Rate of Brain Penetration

Because the rate of drug transfer across the BBB cannot be easily distinguished from its surface area, the BBB permeability for a given drug is instead typically described in terms of a permeability surface area (*PS_in_*) product [68,69], which is measured in units of flow (µL/min/g brain) [14,69]. *PS_in_* is equivalent to the net influx clearance (*Cl_in_*), which can in turn be regarded as the sum of diffusional clearance (*Cl_passive_*) and active uptake transporter clearance (*Cl_uptake_*) of drug across the BBB [70] (Figure 3). It is opposed by the net efflux clearance (*Cl_out_*), which is the sum of diffusional clearance (*Cl_passive_*) and efflux transporter clearance (*Cl_efflux_*) across the BBB, clearance due to drug metabolism at the BBB or in the brain (*Cl_metabolism_*) and clearance due to bulk flow of BECF into CSF (*Cl_bulkflow_*) [70]. The unbound partition coefficient for a given drug at steady-state is entirely determined by the net influx and efflux clearances. If a constant drug concentration in blood is maintained by, e.g., continuous infusion or repeated dosing until the drug has equilibrated across the BBB, *Cl_in_* and *Cl_out_* can therefore be related to *K_p_*_,*uu*,*brain*_ as follows:

(7) $$K_{p,uu,brain}=\frac{Cl_{in}}{Cl_{out}}=\frac{Cl_{passive}+Cl_{uptake}}{CL_{passive}+Cl_{efflux}+Cl_{metabolism}+Cl_{bulkflow}}$$

It can be seen from Equation (7) that the extent of BBB penetration by a drug at steady-state is dominated by active efflux if *K_p,uu,brain_* < 1 (*Cl_out_* > *Cl_in_*), by active influx if *K_p,uu,brain_* > 1 (*Cl_out_* < *Cl_in_*), and by passive transport in both directions if *K_p,uu,brain_* is close to unity (*Cl_out_*~*Cl_in_*) [71,72,73,74]. Provided that active uptake (i.e., *Cl_uptake_*), cerebral drug metabolism (i.e., *Cl_metabolism_*) and drug clearance due to bulk flow of BECF (i.e., *Cl_bulkflow_*) are insignificant compared to passive diffusion (i.e., *Cl_diffusion_*), which is often the case for CNS drugs, Equation (7) can be simplified to

(8) $$K_{p,uu,brain}=\frac{1}{1+Cl_{efflux}/Cl_{diffusion}}$$

In this situation, a high diffusional permeability is desirable to offset the impact of active efflux transport on *K_p,uu,brain_*, as can be seen from the above equation.

There are a number of in vivo or in situ methods that can be used to obtain a measure for the rate of drug transfer from blood to brain [25,48,52,54,57,58,75,76,77,78,79,80] or from brain to blood [54,55,73,81,82,83,84,85,86,87,88] respectively (for reviews see [52,68,89,90]). For example, provided that imaging is paralleled by measurement of radioactivity in plasma, PET measurements and kinetic modeling allow for determination of the parameter *K*_1_ (in mL/min/cm^3^), which corresponds to the rate constant for drug transfer from arterial plasma to the brain [52]. This parameter is similar to the unidirectional transfer constant *K_in_* (in mL/min/g brain) for the initial rate of brain entry, which relates the amount of drug in brain at a given time point after in situ perfusion or in vivo administration in rodents to the amount of plasma exposure up to this point and is calculated according to

(9) $$K_{in}=\frac{A_{tot,brain}}{C_{tot,perf}\times T}=\frac{A_{tot,brain}}{C_{tot,plasma}\times f_{u,plasma}}$$

where *A_tot,brain_* is the total amount of drug per g of brain tissue (corrected for remaining drug in the vasculature), *C_tot,perf_* and *T* are the concentration of drug in the perfusate and the net perfusion time (for in situ techniques), and *C_tot,plasma_* and *f_u,plasma_* are the area under the drug concentration-time curve and the unbound drug fraction in plasma (for in vivo techniques) [61,68,91].

*PS_in_* can be calculated from *K_in_* or *K*_1_ based on the basic principles of the capillary flow model using the Renkin–Crone equation [92,93]

(10) $$PS_{in}=-F\times ln\left(1-\frac{K_{in}}{F}\right)=-F\times ln\left(1-\frac{K_{1}}{F}\right)$$

where *F* is the rate of perfusion (for in situ techniques) or CBF (for in vivo techniques) [61,91]. For most CNS drugs, *F* is much larger than *PS_in_* (*F* > 5 × *PS_in_*), so that CBF is not rate-limiting and *K_in_* or *K_1_* equal *PS_in_* with less than 10% error. If *PS_in_* is much larger than *F* (*PS_in_* > 2.3 × *F*) on the other hand, CBF becomes rate-limiting for drug delivery into the brain and *K_in_* or *K*_1_ equal *F* with less than 10% error [91].

Provided that CBF is not rate-limiting, *PS_in_* gives a measure for the initial rate of drug transfer across the BBB, but the actual time required for full equilibration of various drugs between blood and brain has been shown to also depend on their tendency for non-specific binding to brain tissue [15,51,63,94]. Based on experimental data and pharmacokinetic modeling, the time to achieve brain equilibrium has therefore been described in terms of an intrinsic brain equilibrium half-time *t*_1/2*eq,in*_

(11) $$t_{1/2eq,in}=\frac{ln2\times V_{b}}{PS_{in}\times f_{u,brain}}$$

where *V_b_* is the physiological volume of brain tissue [94]. Since *V_b_* is a constant, Equation (11) indicates that not the rate of BBB penetration per se but rather the product of *PS_in_* and *f_u,brain_* for a compound determines the time to reach brain equilibrium, which is supported by a number of theoretical calculations and experimental findings [63,72,94,95]. Thus, as described in more detail elsewhere [72,94], lipophilic compounds often show high rates of passive transfer across the BBB but also a high degree of non-specific binding, so that their *t*_1/2*eq,in*_ may be very similar to that of less lipophilic compounds with low rates of passive transfer and a low degree of non-specific binding.

An alternative, minimally invasive method for in vivo assessment of BBB permeability in animal models is based on laser-scanning multiphoton fluorescence microscopy of the cerebral microcirculation through a thinned section of the skull bone [96,97,98]. This approach involves infusion of fluorescent solutes into the cerebral circulation and simultaneous collection of temporal images to quantify their rate of tissue accumulation and radial concentration gradient around individual microvessels [96]. Using a suitable mathematical model and taking into account the influence of solvent drag and other factors, these parameters can be used to directly derive the apparent in vivo permeability *P* (in cm/s) for transfer of the test compound from vessel lumen to brain as well as its effective diffusion coefficient *D_eff_* (in cm^2^/s) in brain tissue [96,99]. Another distinct advantage of this technique over most alternative approaches is that it provides subcellular level details and allows for in vivo studies on region-specific differences in BBB permeability under physiological conditions, which are likely to exist but still poorly characterized [100].

Nevertheless, due to the time-intensive and costly nature of in vivo or in situ methods for determination of *P* or *PS_in_*, BBB penetration rates of candidate drugs are much more frequently measured in in vitro models of the BBB [90]. To this end, the drug of interest is added to one of two compartments separated by the model BBB on a special membrane (for details see Section 4), its concentration in both compartments is repeatedly measured over time and used to calculate the incremental clearance volume Δ*V_CL_* for each time point according to

(12) $$\Delta V_{CL}=\frac{C_{receiver}\times V_{receiver}}{C_{donor}}$$

where *C_donor_* is the concentration in the compartment to which the drug has been added while *C_receiver_* and *V_receiver_* are the concentration in and volume of the compartment into which the drug is cleared respectively. Provided that drug concentrations in the receiver compartment remain small and Δ*V_CL_* increases in a linear fashion, the slope of the line obtained by plotting Δ*V_CL_* as a function of time provides a measure of the total PS product *PS_t_* (in µL/min) for unidirectional transfer across the model system (i.e., endothelium and cell-free membrane). It can be converted to the PS product *PS_e_* (also in µL/min) for unidirectional transfer across the endothelium according to

(13) $$\frac{1}{PS_{e}}=\frac{1}{PS_{t}}-\frac{1}{PS_{m}}$$

where *PS_m_* (in µL/min) is the PS product for transfer across the cell-free membrane that separates the two compartments. Finally, *PS_e_* can be converted to the in vitro permeability coefficient *P_e_* (in cm/min) according to

(14) $$P_{e}=\frac{PS_{e}}{S}$$

where *S* is the known exchange surface area of the model BBB (in cm^2^).

Alternatively, an apparent in vitro permeability coefficient *P_app_* (in cm/s) is often calculated according to

(15) $$P_{app}=\frac{J}{C_{donor\left(o\right)}\times S}$$

where *J* is the rate of appearance of the drug in the receiver compartment (in s^−1^) and *C_donor_*_(0)_ is the drug concentration in the donor compartment at the start of the experiment (in mL^−1^). Because *P_app_* reflects the combined permeability across the endothelium and the cell-free membrane, it is related to *P_e_* according to

(16) $$\frac{1}{P_{app}}=\frac{1}{P_{e}}-\frac{1}{P_{m}}$$

where *P_m_* is the permeability coefficient across the cell-free membrane.

Finally, because in vitro models allow for measurement of BBB transfer into both directions, they can also be used to determine a bi-directional efflux ratio (*ER*) according to

(17) $$ER=\frac{P_{e\left(AL\right)}}{P_{e\left(LA\right)}}=\frac{P_{app\left(AL\right)}}{P_{app\left(LA\right)}}$$

where *P_e(AL)_* and *P_app(AL)_* are the permeability coefficient and apparent permeability coefficient for unidirectional transfer from abluminal to luminal compartment (i.e., “brain to blood”) and *P_e(LA)_* and *P_app(LA)_* are the permeability coefficient and apparent permeability coefficient for unidirectional transfer from luminal to abluminal compartment (i.e., “blood to brain”), respectively (unless stated otherwise, the terms *P_e_* and *P_app_* in the rest of this article will refer to *P_e(LA)_* and *P_app(LA)_*). For a perfect in vitro model that accurately reproduces all passive and active transport properties of the BBB in vivo and also takes into account metabolism and elimination via BECF bulk flow, ER should correspond to 1/*K_p,uu,brain_*. However, as described in the following sections, such models do neither exist, nor are they likely to become available in the near future, so that reliable prediction of *K_p,uu,brain_* from in vitro determined efflux ratios is not feasible.

### 3.3. Implications for CNS Drug and Neurotracer Development

Taken together, the extent of drug entry into the brain can be affected by drug binding to plasma proteins, by the distribution of the drug in the brain, by passive and active transport processes at the BBB and by clearance of the drug via metabolism or bulk flow of BECF into CSF (Figure 3). In contrast, the rate of brain entry is either determined by the rate of BBB penetration (i.e., the sum of passive drug diffusion and active drug uptake across the BBB) or by drug delivery via CBF, with the slower process being rate limiting. Finally, the time required for complete drug equilibration between blood and brain depends not only on the rate of brain entry, but also on the degree of non-specific drug binding to brain tissue. As such, a comprehensive description of drug delivery to and distribution within the brain requires knowledge of at least three parameters, namely, *K_p,uu,brain_*, *PS_in_*, *V_u,brain_* and/or *f_u,brain_*.

With regard to drugs that are dosed continuously, the extent of brain penetration (i.e., *K_p,uu,brain_*) is now generally regarded as the most important parameter for identification of successful CNS-targeted therapeutics, while *PS_in_* seems to be much less important and can differ between CNS-active drugs by a factor of up to at least 20,000-fold [14]. This range is several orders of magnitude larger than that for *K_p,uu,brain_*, which only differs by a factor of up to 150-fold [14], making it a much better descriptor of drug delivery to the brain. Preferably, the *K_p,uu,brain_* for a CNS-targeted drug should be close to unity, since plasma and brain concentration-time profiles for such compounds tend to run in parallel and unbound brain concentrations are equal to the product of *f_u,plasma_* and *K_p,uu,brain_*. Likewise, compounds with *ER* values below 2.5–3.0 and ideally around unity are generally regarded as promising candidates for further evaluation [61,101,102,103]. However, one should keep in mind that *ER* values are derived from in vitro models, which are still most frequently based on non-BBB cells transfected with the major efflux transporter MDR1 (see Section 4.2.1). As even more complex in vitro models invariably fail to reproduce all transport properties of the BBB in vivo (see Section 4), *ER* values can at best provide an upper limit for the true value of 1/*K_p,uu,brain_*. Thus, while a high *ER* points to active efflux of drug across the BBB, which will most likely also reduce brain penetration in vivo, a low *ER* may simply reflect the fact that the BBB model used lacks the relevant efflux transporter. As such, current in vitro models have limited value for predicting the actual extent of brain penetration in vivo, but they can facilitate early identification of substrates for important efflux transporters, at least if these transporters are expressed in the particular model system used [61,89,102].

The distribution of drug in brain is best described by *V_u,brain_*, which should be in the order of 0.2 mL/g if the drug target is accessible from BECF, or not much larger than 0.8 mL/g if the drug target is accessible from BICF [15]. If the aim is to develop fast-acting CNS drugs or neurotracers labeled with short-lived radionuclides, the rate of brain entry becomes important as well. Since determination of in vivo or in situ *PS_in_* values is time-intensive and costly, BBB permeability measurements are often performed in vitro. Provided that CBF is not rate-limiting for drug delivery and active uptake of drug into the brain can be neglected, the initial rate of brain penetration should be entirely determined by passive diffusion across the BBB. In this case, permeability estimates obtained in in vitro BBB models with adequate physical barrier properties should be much less prone to error than *ER* values, even if the model used fails to reproduce all active transport processes of the BBB in vivo. If active drug transport from blood to brain cannot be excluded, in vitro permeabilities should still provide a lower limit that, at the worst, underestimates the true rate of BBB penetration in vivo. In general, compounds exhibiting in vitro *P_app_* values of at least 0.5–1.0 × 10^−5^ cm/s (again most commonly measured using models based on non-BBB cells transfected with the efflux transporter MDR1, see Section 4.2.1) are regarded as promising candidates for fast acting drugs or neurotracers [61,101,102,103]. However, because the time to brain equilibration as well as the usefulness of a compound for PET imaging are also determined by non-specific brain tissue binding, *f_u,brain_* for a fast-acting CNS drug or neurotracer should be sufficiently high (>0.05 and preferably >0.15) as well, while *V_u,brain_* should not exceed 0.2–0.8 mL/g (depending on the drug target as described above), in which case, even compounds with a low BBB permeability can be useful candidates. With regard to receptor-targeted neurotracers, the maximum concentration of binding sites (*B_max_*) in the brain and tracer affinity for these sites (*K_d_*), which can be determined by saturation binding assays or in vitro autoradiography studies, are additional design criteria, since imaging of targets with a low *B_max_* requires ligands with a higher affinity (i.e., lower *K_d_*) [103]. In general, the ratio of these parameters (*B_max_*/*K_d_*) for successful neurotracers should be ≥10, which means that ligands with sufficient affinity for a target with low expression level (*B_max_* < 1 nM) can be challenging to identify, because they should also be selective for the target (preferably >30–100×) and comply with the other criteria for fast-acting drugs [103,104]. Likewise, receptor-targeted neurotracers should either be metabolically stable or their metabolism should be confined to the periphery and any radiometabolites formed should be unable to cross the BBB, as separation of brain radioactivity arising from a parent compound and its metabolites is usually very difficult if not impossible to achieve [105].

Finally, in closing this section, it should be noted that a number of (experimentally determined or more commonly calculated) physicochemical properties have been shown or are thought to correlate with BBB permeability and/or the non-specific binding behavior of CNS drugs, so that they are frequently used for a first in silico ranking of candidate compounds, especially if pre-existing in vitro or in vivo data is lacking [53,106]. These properties most commonly comprise the logarithm of the calculated or measured partition coefficient between octan-1-ol and water (clogP or logP), the logarithm of the calculated or measured distribution coefficient between octan-1-ol and a buffer—usually, 0.1 m sodium phosphate buffer—at pH 7.4 (clogD or logD), the molecular weight (MW), the topological polar surface area (TPSA), the ionization constant of the most basic center (pKa), and the number of hydrogen bond donor atoms (HBD). As described in more detail elsewhere [103,107,108], multi-parameter optimization (MPO) approaches can be used to obtain a single weighted score (CNS MPO or CNS PET MPO score) that incorporates all of these physicochemical properties. If compared to predictions based on individual parameter values, CNS MPO scores ≥ 4.0 or CNS PET MPO scores ≥ 3.0 have been demonstrated to increase the probability of identifying compounds that combine suitable values of *P_app_*, *ER* and *f_u,brain_* and to provide improved differentiation between successful and failed CNS drugs or neurotracers, respectively [103,106,107,108,109].

In addition, some groups have employed empirical in silico models based on large compound libraries for early estimation of *P_app_*, *ER* and *f_u,brain_* during ligand design and lead prioritization [103,109,110]. The reliability of such estimates will obviously depend on the structural similarity between a candidate drug or neurotracer and compounds included in the training data for the model. Especially if experimental values for a sufficient number of compounds from the same chemical series are available, it may therefore be worthwhile to use them as a basis for model-based in silico lead prioritization during the development of novel CNS therapeutics or neurotracers.

## 4. In Vitro Models of the BBB

Since the first isolation of brain capillaries in the early 1970s, a number of in vitro BBB models with variable complexity have been developed. Not least due to the highly dynamic nature of the BBB, which is still incompletely understood, none of these models exactly mimics the in vivo conditions, and all of them suffer from certain limitations and drawbacks. As such, the choice of a model should be closely matched to the exact requirements of a given study and any findings should be interpreted in view of these limitations. In general, because current in vitro models invariably overestimate the permeability of the BBB in vivo, they seem to be most useful for early identification of poorly BBB penetrating compounds and (for models that incorporate efflux transporters like PGP/MDR1) compounds that are likely to be subject to active efflux. The following sections will provide an overview of the advantages and limitations of the most commonly used BBB models, which can be roughly classified into cell-free, cell-based static and cell-based dynamic approaches.

### 4.1. Cell-Free Model Systems

A frequently used cell-free approach to evaluate the passive diffusion properties of CNS-targeted drugs in the pharmaceutical industry is the parallel artificial membrane permeability assay (PAMPA), which was originally developed to predict drug uptake in the gastrointestinal tract [111]. For this technique, a filter-supported artificial membrane that can be composed of a variety of phospholipid mixtures is used to separate two buffer-containing compartments, the drug is dissolved into one of the compartments and then allowed to permeate through the artificial membrane. PAMPA models based either on porcine brain lipid extract dissolved in *n*-dodecane (PAMPA-BBB) or on black lipid membranes (PAMPA-BLM) have been shown to appropriately identify a number of structurally diverse drugs as either BBB permeable or non-permeable [112,113,114,115,116]. Importantly, many of these drugs have also been shown to be roughly 1–2 orders of magnitude less permeable in PAMPA models with phospholipid mixtures that match the composition of human BMEC membranes, suggesting that species-specific lipid models are preferable for passive permeability assays [117]. Major advantages of PAMPA-based models are their very low cost and technical simplicity, a high degree of reproducibility and the fact that they can be performed in multi-well plates and used for high-throughput screenings [111,112]. The most obvious disadvantage is a complete lack of BBB-specific transporters, so that these models are only useful to evaluate the passive permeation properties of CNS-targeted drugs.

Another cell-free approach for rapid assessment of passive permeation properties is immobilized artificial membrane (IAM) chromatography, which involves covalent bonding of synthetic lipid analogs to the surface of silica particles, which are then used as packing material for a high-performance liquid chromatography (HPLC) column [118,119,120]. The idea behind this technique is that drug permeation across the cell membrane is limited by the drugs ability to partition into the lipid domain. Because drugs which interact with the lipid phase have longer retention in the IAM column, large capacity factors are regarded as an index for good permeability across lipid bilayers. Although the retention times obtained by IAM chromatography do not reflect actual drug passage across a membrane, they have been shown to generate reasonable predictions of passive membrane permeability [121,122]. The advantages and disadvantages of IAM chromatography are similar to those of PAMPA-based approaches: the technique is rapid, cost efficient and technically simple but only provides information about passive transport processes.

### 4.2. Cell-Based Static Models

**Figure 4:**
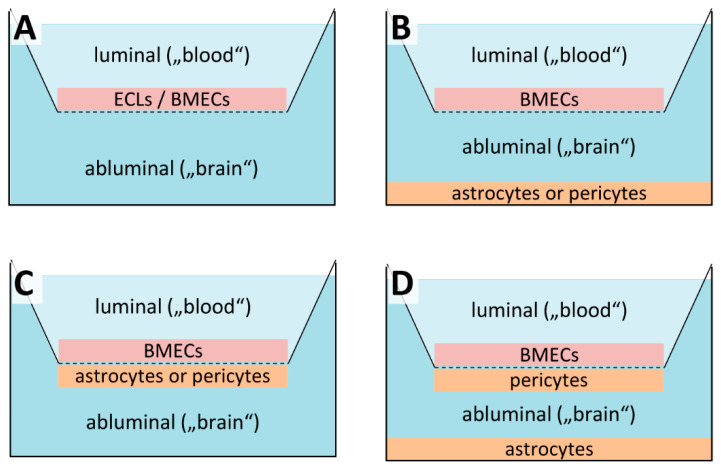
Static in vitro models of the blood-brain-barrier (BBB). (**A**) Simple static models are typically either based non-BBB epithelial cell lines (ECLs) that may be transfected with BBB-specific efflux transporters or on primary/immortalized brain microvascular endothelial cells (BMECs). The cells are grown as a monolayer on microporous, semipermeable membrane-based cell culture inserts that separate a cell culture well into luminal and abluminal compartment for permeability assays. (**B**) For static non-contact co-culture models, astrocytes or, less frequently, pericytes are grown on the bottom of the cell culture well to allow for indirect cell-to-cell communication with the BMECs via secreted soluble factors. (**C**) Static contact co-culture models are similar to non-contact models, except that the second cell type is grown on the underside of the cell culture insert and thus in close proximity of the BMECs. (**D**) For static triple co-culture models, pericytes are grown on the underside of the cell culture insert while astrocytes are grown on the bottom of the culture well in order to more closely resemble the multicellular nature of the BBB in vivo.

Owing to their relatively low cost and technical simplicity, static BBB models that do not imitate the shear stress generated by CBF remain the most widely used cell-based systems. For these models, a monolayer of cells is grown on the upper side of a microporous (0.2–0.4 µm pore size) semipermeable membrane-based cell culture (Transwell^®^) insert that allows exchange of small molecules but prevents cell migration. The insert mimics the blood (luminal) side of the BBB and is placed into a cell culture well that mimics the parenchymal (abluminal) side (Figure 4A). Monolayer tightness can be quantified in terms of the TEER and/or the permeability to different paracellular permeability markers, which are small polar molecules that lack measurable active transport and typically comprise (radiolabeled) sugars like sucrose (MW 342, r 4.6 Å) or mannitol (MW 182, r 3.6 Å), and fluorescent dyes like sodium fluorescein (MW 376, r 4.5 Å) or Lucifer Yellow (MW 443, r 4.2 Å) [10]. Although TEERs can be affected by a number of factors and direct comparison between different studies is difficult (for reviews see [123,124]), a value of 150–200 Ω × cm^2^ is generally regarded as the lower limit for useful in vitro models [125]. Likewise, in vitro permeability for the small polar tracer molecules described above should be within a factor of 100 of the in vivo range, which does not usually exceed values corresponding to a *P_app_* of roughly 1.4 × 10^−7^ cm/s [24,25,26]. While these parameters provide a measure for the physical barrier properties of a model, assessment of its ability to reproduce active transport processes of the BBB in vivo requires detailed analysis of transporter expression levels and is highly dependent on the drugs of interest. Due to a lack of standardization with regard to cell isolation and culture conditions, transporter expression levels may significantly vary between studies, even if the same model system and cell types are used. For this reason, they will not be covered in detail in the present article, which will instead provide a general overview of the different cell-based BBB models. As described below, static models range from simple approaches based on genetically-modified epithelial cell lines for use in high-throughput screenings to more elaborate co-culture systems that incorporate multiple, CNS-derived primary or immortalized cell types to more closely recapitulate the unique barrier properties of the BBB in vivo. Another potential source of BMECs and other CNS cells for use in in vitro BBB models that has been reviewed in detail elsewhere are human pluripotent stem-cells [126,127,128,129,130].

#### 4.2.1. Monolayer Models Based on Non-BBB Cells

In their simplest form, static monolayer BBB models may be based on an immortalized epithelial cell line overexpressing one or more of the transporters found in BMECs. For example, one of the first and most widely used models for high-throughput ranking of BBB permeability in the pharmaceutical industry is based on the Madin–Darby Canine Kidney (MDCK) cell line [63,102,131], which was originally derived from the kidney of an adult female cocker spaniel. Especially if transfected with MDR1, an efflux pump known to be highly active at the BBB in vivo [63], MDCK cells form a tight monolayer with permeability values for various compounds that are in reasonable agreement with in vivo brain permeation, even though TEERs do not usually exceed 200–300 Ω × cm^2^ (Table 1). In addition, assays with MDCK cells can be easily automated and used for rapid generation of permeability data for a large number of compounds.

**Figure 5:**
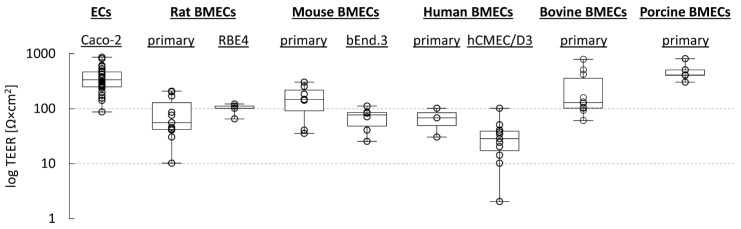
Differences in reported TEER values for monolayer cultures based on different cell types. Shown are individual values taken from the literature (open circles, for references see Table 1) and boxplots constructed from the median value, upper and lower quartiles (box) and minimum and maximum values (whiskers). Monolayers grown from epithelial cells (ECs) like the Caco-2 cell line show relatively high TEERs but lack other features of brain microvascular endothelial cells (BMECs). In contrast, monolayers grown from primary or immortalized (RBE4, bEnd.3 and hCMEC/D3) BMECs seldom exceed 300 Ω × cm^2^, with the exception of bovine and porcine primary cells. Note that data from publications that used hydrocortisone to increase TEERs have been excluded to facilitate comparison between the different species/cell types.

Another widely used cell line is Caco-2, an intestinal epithelial cell line derived from a human colon adenocarcinoma. Although primarily used for the prediction of small intestine drug absorption [132], Caco-2 cells have been shown to give acceptable predictions of BBB penetration for passive diffusion compounds [9,133] and, if treated with vinblastine, to increase MDR1 expression (VB-Caco-2 [134]), also for ligands of this efflux transporter [133]. In most studies with Caco-2 cells, TEERs were in the order of 250–500 Ω × cm^2^, but values as low as 86 Ω × cm^2^ and as high as 800 Ω × cm^2^ have been reported as well (Figure 5, Table 1), possibly reflecting differences in measurement technique, passage number, culture conditions and/or other factors. Likewise, *P_app_* values for penetration of small polar solutes (usually mannitol) through Caco-2 monolayers obtained in different laboratories cover a relatively broad range (Table 1), complicating strict comparisons between studies.

Given the structural and functional differences between epithelial and endothelial cells and the complete lack of a BBB microenvironment however, it is evident that models based on non-BBB cells have little value for accurate prediction of in vivo net transport rates into the CNS. Nevertheless, they represent very attractive systems for a first, rapid and cost-efficient ranking of large compound libraries with regard to passive diffusion properties (and MDR1-mediated efflux), not least because their tightness is usually superior if compared to simple monolayer models based on primary or immortalized BMECs (Figure 5, see also Section 4.2.2). As described in the following subsections, subsequent validation of promising compounds in co-culture models based on BMECs is still advisable, since these models typically outperform non-BBB cell-based models for several reasons. Since even the most complex models currently available invariably fail to accurately reproduce all active transport processes at the BBB in vivo, compounds with promising diffusion properties may be further evaluated using cells expressing particular transporters or any other suitable assay for identification of efflux transporter substrates.

#### 4.2.2. Monolayer Models Based on BMECs

To more closely recapitulate the unique barrier properties of the BBB, a number of studies have used monolayer models based on primary or low passage number BMECs obtained from mouse [199,200,201,205], rat [190,194,195,196,197,198], porcine [212,213,214] or bovine [215,216,217,218,219,220,223,230,231,232] brain. Due to the restricted availability of human material for cell isolation, only a few studies have been performed with primary BMECs of human origin [216,233,234,235].

Regardless of the exact species used, a major disadvantage of primary cells that has prevented their routine application in the drug development setting is that isolation of BMECs is technically challenging and associated with a high risk for contamination by mural cells [10,236]. In addition, BMECs account for only 0.1% (*v/v*) of the brain, so that a large number of animals may be required to obtain a sufficient number of cells, especially in the case of rodent studies [130]. The latter can be partly overcome by the use of BMECs from larger species, which has the added advantage that TEERs are usually higher (Figure 5) but precludes the use of genetically modified animals. Finally, primary BMECs may show considerable batch to batch variations [9,224] and can only be cultured for a limited time, during which the lack of in vivo environmental cues may result in uncontrolled changes of transporter expression and/or tightness of the endothelium. Thus, while the expression pattern of freshly isolated primary cells typically closely matches the unique phenotype of BMECs in vivo, this conformity quickly diminishes over time in culture [9,237]. As such, considerable research efforts have been devoted to the establishment of suitable immortalized BMEC cell lines, which are often more stable in their endothelial traits and could significantly reduce the cost and technical expertise required to establish in vitro BBB models [237,238].

At present, at least 36 different immortalized BMEC lines have been described and used for in vitro BBB models (reviewed in [238]), with the most common ones being the human hCMEC/D3, the rat RBE4 and the mouse bEnd.3 cell line. The latter three cell lines are relatively well characterized and have been shown to retain important BBB characteristics, such as the expression of TJs and certain efflux transporters [161,176,182,238,239,240,241].

However, as illustrated in Figure 5 and Table 1, TEER values for static monolayer models with immortalized BMEC lines are usually much lower than those obtained with epithelial cell lines, while the permeability for common tracer molecules is often higher [172,176,182,238,242,243]. This may in part reflect the lack of input from other CNS cell types, since monolayer models based on primary BMECs from the same species typically show similar TEERs (Figure 5) and apparent permeabilities (Table 1). In general, somewhat higher TEERs can be achieved in models based on primary porcine or bovine BMECs, which has been proposed to reflect differences in cell size and/or the complexity of TJ organization [191]. Another potential explanation is that contamination by mural cells, which can adversely affect monolayer tightness, is simply more difficult to avoid when BMECs are isolated from a small species like mouse or rat. Interestingly however, a similar variation between species has not always been observed with regard to *P_app_* values for permeability markers like mannitol, suggesting that differences in TEER may not necessarily translate to differences in permeability of the endothelial monolayers [191].

Approaches to overcome the limitations of monolayer models with a single cell type include the use of serum-free, astrocyte-conditioned medium, addition of BBB modulating compounds like glucocorticoids and coating of the culture surfaces with ECM components like collagen or laminin to obtain tighter endothelial phenotypes [213,216,244,245]. These techniques may partly substitute for the lack of cell-to-cell communication, and especially the addition of compounds like hydrocortisone has in some cases been reported to result in TEERs as high as 600–1000 Ω × cm^2^ and sucrose permeabilities as low as 3 × 10^−7^ cm/s [216,245]. However, the absence of other CNS cell types can also adversely affect the performance of monolayer cultures through factors that depend on physicochemical properties of the tested drugs. This has recently been illustrated by comparison of the in vitro permeabilities of 27 marketed CNS drugs, as determined in a bovine monolayer model, with the corresponding permeabilities determined in vivo [246]. While there was a strong correlation for hydrophilic compounds with low brain tissue binding, the correlation for lipophilic compounds with high brain tissue binding was poor [246]. Co-culture with glial cells to mimic brain tissue and incorporation of binding to these cells in the in vitro calculations on the other hand resulted in a strong correlation between in vitro and in vivo permeabilities for the whole set of compounds [246]. These results are in line with previous findings that the time to brain equilibration by lipophilic drugs can be strongly affected by their ability to bind to brain tissue [51,63,72,94,95]. Monolayer models based on BMECs as the only cell type may therefore be of limited value for prediction of in vivo BBB penetration by lipophilic compounds, which represent the majority of CNS-penetrant drugs. Better results may be obtained with systems that are based on co-culture of BMECs with other CNS cells, as described in the next section.

#### 4.2.3. Co-Culture Models Based on BMECs

Based on recognition that other CNS cell types are critically involved in the maintenance and regulation of BBB function in vivo [5,36,42,199,244,247], a number of co-culture BBB models have been established. They can be roughly classified into (i) non-contact double co-culture models, where another type of CNS cells (astrocytes or less frequently pericytes) is grown on the bottom of the culture well (Figure 4B) [190,193,194,212,248,249,250,251], (ii) contact double co-culture models, where the other type of CNS cells is grown on the underside of the insert (Figure 4C) [193,194,212,218,220,251], and (iii) triple co-culture models, where pericytes are grown on the underside of the insert and astrocytes are grown on the bottom of the culture well (Figure 4D) [173,194,196,229,252].

**Figure 6:**
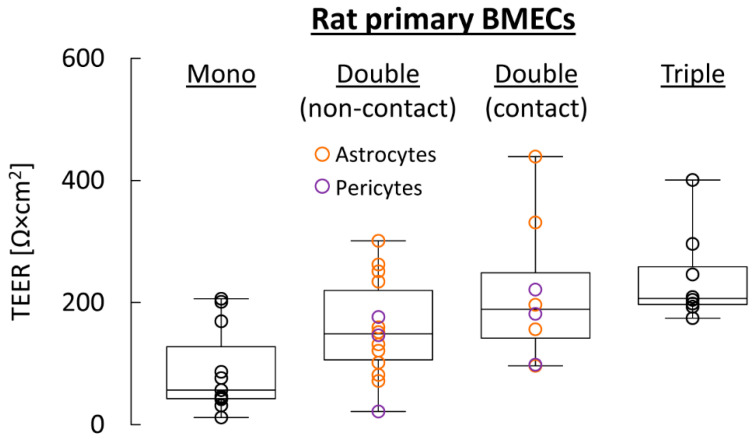
Effect of culture conditions on reported TEER values for static BBB models based on rat primary cells. Shown are individual values taken from the literature (open circles, for references see Table 1) and boxplots constructed from the median value, upper and lower quartiles (box) and minimum and maximum values (whiskers). As described in more detail in the main text, TEER values reported for non-contact co-cultures of rat brain microvascular endothelial cell (BMECs) with astrocytes or pericytes are usually about two-fold higher than the corresponding values for monolayer cultures of BMECs grown in the absence of other BBB cells. Even higher TEERs may be achieved by contact co-culture with either astrocytes or pericytes or by co-culture of all three cell types. Note that data from publications using hydrocortisone to increase TEERs have been excluded to facilitate comparison between the different culture conditions.

While the cost and technical expertise required for double or triple co-culture models are higher than for the simpler monolayer approach, the ability for indirect cell-to-cell communication via secreted soluble factors has been shown to promote an endothelial phenotype that much more closely resembles the BBB in vivo, primarily by inducing cell polarity in BMECs and increasing the expression of transporters and TJs [194,196]. Thus, TEERs achieved with double or triple co-culture models based on primary cells can be as high as 780 Ω × cm^2^ for rodent BMECs (Figure 6, Table 1) or 2500 Ω × cm^2^ for BMECs from larger species respectively (Table 1). Likewise, the permeability towards common paracellular permeability markers has been shown to be reduced if compared to simple monolayer models (Table 1). In addition, comparison of in vitro data for a number of compounds obtained in different co-culture models with in vivo data obtained using PET imaging revealed good to excellent correlations, while there was no correlation of the in vivo results with in vitro data obtained using Caco-2 cells or with various physicochemical properties (for details see Table A1 in Appendix A) [253,254,255]. Finally, routine application of these models is facilitated by the commercial availability of ready-to-use solutions like the BBB Kit™ triple co-culture system [256], which offers the choice between primary cells from a range of species. As such, they represent useful systems for small- to moderate-scale studies on BBB penetration that are much less cost- and labor-intensive than most of the dynamic BBB models described in the following subsections. Even though the true rate of brain entry may still be overestimated by these models, they could facilitate early detection of slowly penetrating compounds that are unlikely to be useful as fast-acting drugs or imaging probes.

### 4.3. Cell-Based Dynamic Models

Dynamic BBB models derive their name from the fact that they incorporate shear stress to simulate the effects of CBF, which further improves barrier function, reduces permeability and results in an endothelium that more closely resembles the in vivo properties of the BBB. As described in the following subsections, these models can be further separated into the cone-plate BBB apparatus, conventional dynamic and microfluidic-based dynamic models.

#### 4.3.1. The Cone-Plate BBB Apparatus

**Figure 7:**
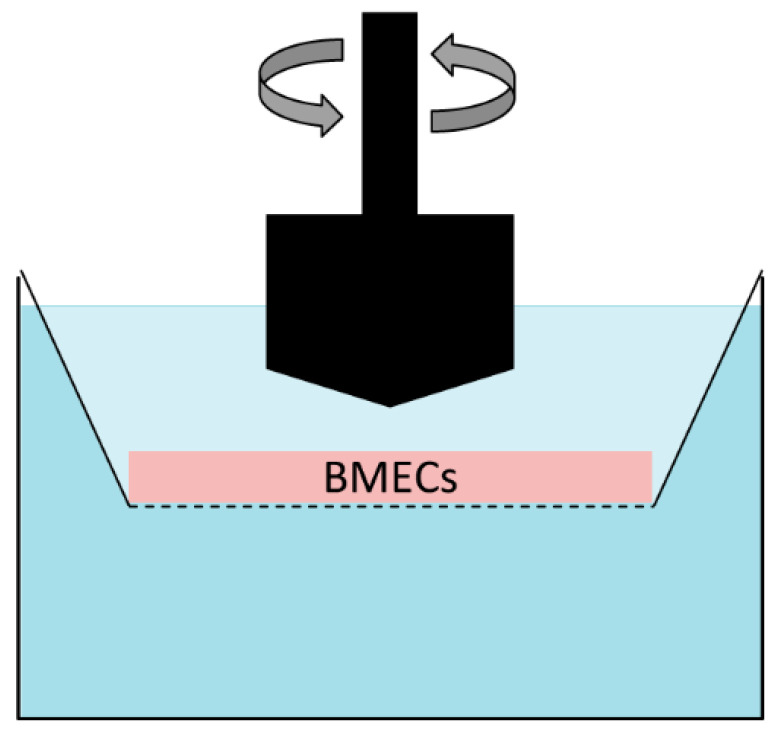
The cone–plate blood–brain barrier (BBB) apparatus. As a simple approach to simulate the effects of cerebral blood flow in static monolayer models, the cone-plate BBB apparatus uses a rotating cone to produce shear forces that reach the endothelial cells through the culture medium. This technique has limited reliability and is not widely used for BBB studies.

One early approach that can be used to simulate the effects of CBF in models based on monolayer cultures of BMECs involves application of the cone-plate BBB apparatus, a device with a rotating cone used to produce shear forces that reach the endothelial cells through the medium (Figure 7) [257,258]. The level of shear stress thus produced is determined by cone angle and angular velocity, while its nature (i.e., laminar or pulsatile) depends on the exact mode of operation. However, this approach has limited reliability and is not widely used for BBB studies, since the resulting shear stress is not uniformly dispersed along the radius of the monolayer and differs markedly from the shear stress produced by CBF in vivo [8,259].

#### 4.3.2. Dynamic in Vitro (DIV) Models

**Figure 8:**
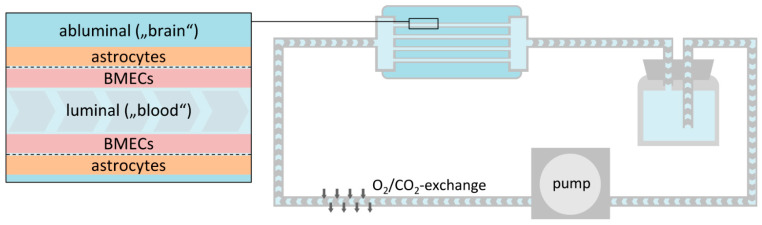
Dynamic in vitro (DIV) models of the blood–brain barrier (BBB). DIV BBB models use a variable-speed pulsatile pump to push culture medium through a co-culture of brain microvascular endothelial cells and astrocytes located in the inner and outer side of microporous hollow fibers respectively. The resulting shear stress simulates the effects of cerebral blood flow in vivo, thereby improving barrier function and expression of BBB-specific transporters when compared to static co-culture systems.

Most dynamic in vitro (DIV) BBB models are instead based on co-culture of BMCEs and astrocytes in the inner (luminal) and outer (abluminal) sides of microporous hollow fibers (Figure 8), which may optionally feature transmural microholes for transmigration or trafficking studies [172,181,187,260,261,262]. To simulate CBF, a variable-speed pulsatile pump connected to the system by gas-permeable tubing (for exchange of O_2_ and CO_2_) is used to push the culture medium through the hollow fibers, which results in intraluminal pressures and shear stress very similar to those under physiological conditions. Due to their experimental nature and limited commercial availability (however, see [263]), the number of studies performed with these systems is still small when compared to static BBB models (Table 1). The potential importance of shear stress for accurate reproduction of the in vivo properties of the BBB is illustrated by the fact that co-culture of hCMEC/D3 cells with astrocytes in a dynamic model gave TEERs in the order of 1000 Ω × cm^2^, while the TEERs obtained by co-culture of the same cells in a static system only amounted to 70 Ω × cm^2^ [172]. Nevertheless, the requirement for a much larger number of cells (>10^16^) when compared to static co-culture models and the elaborate setup required for these models render their application for high-throughput screening unfeasible. In addition, the time required for steady-state TEERs to be reached (9–12 days) [172,181,187,260,261,262] is usually longer than that for static monolayer models (3–4 days) [190,194,195,196,197,198,199,200,201,205]. As briefly described in the following subsection, the development of microfluidic-based systems that also produce shear stress to simulate the effects of CBF and are suitable for high-throughput screens could represent an important step towards the more widespread use of dynamic BBB models for drug development purposes.

#### 4.3.3. Microfluidic-Based Dynamic Models

**Figure 9:**
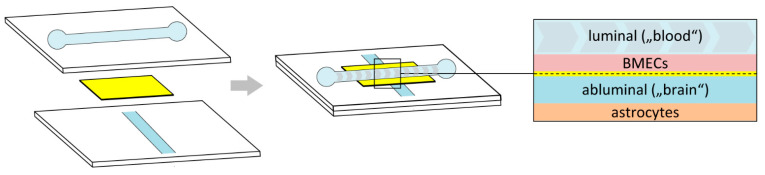
Microfluidic-based dynamic blood–brain barrier (BBB) models. Most microfluidic-based dynamic BBB models integrate the monolayer of brain microvascular endothelial cells (BMECs) into a planar, microfluidic network that allows for co-culture with one or more additional cell types and introduction of fluid flow-induced shear stress.

Microfluidic-based BBB models are similar to DIV BBB models in that they simulate the shear forces generated by CBF in vivo, but they have several potential advantages that could facilitate their use in high-throughput screenings. Although the exact design of microfluidic models can vary, they are typically composed of two small channels for co-culture of BMECs and astrocytes or pericytes that are separated by a polycarbonate porous membrane placed over their intersection [151,164,189,192,264] (Figure 9). The much smaller size of the channels compared to the porous hollow fibers used in classic dynamic models not only better replicates the in vivo microcirculatory system, but also significantly reduces the number of cells required, allows for more precise measurement of TEERs, and reduces the time to reach steady-state TEERs to 3–4 days [151,164,189,192,264]. In general, TEERs in the order of 600–1000 Ω × cm^2^ have been reported for rodent primary BMECs [151,192] as well as for immortalized mouse bEnd.3 [189] or human hCMEC/D3 [175] cells grown in microfluidic systems, although much lower values were observed in some other studies [151,164] (Table 1). The latter could in part be related to the use of different systems and/or culture conditions, or to the fact that only two types of cells can be co-cultured in most (but not all) current microfluidic-based BBB models. In any case, with a few exceptions (like the SynVivo platform [265]), current microfluidic models are not commercially available, and the inherent complexity of fabricating and operating these systems restricts their use to laboratories with significant bioengineering expertise. Given further refinement and better commercial availability of these models, however, it seems likely that they could become the systems of choice for high-throughput studies on BBB permeability of drugs and imaging probes.

## 5. Conclusions

Taken together, a number of in vivo, in situ and in vitro techniques have been used to measure or predict drug penetration across the BBB. Although robust estimates for the rate of BBB penetration can often be obtained with suitable in vitro BBB models, they are now recognized as poor predictors for the success of CNS drugs that are dosed continuously. However, they remain useful measures for the development of fast-acting drugs or neurotracers, especially if they are complemented with data on non-specific drug binding to brain tissue. In many cases, in vitro models can also be used to screen for drugs that are subject to active transport (i.e., influx or efflux) across the BBB, although their predictive value in this regard is much less robust and can be highly dependent on the drugs of interest. Thus, despite significant progress in the understanding of BBB structure, function and regulation, current in vitro models invariably fail to accurately reproduce all active transport properties of the BBB in vivo. Whereas microfluidic-based dynamic models promise to more closely reflect the in vivo microcirculatory system, most of these models are still in the proof-of-principle phase and their application is limited to laboratories with significant bioengineering expertise. The same applies to more complex three-dimensional models of the brain microvasculature, which are still in development and have not been addressed in the present article, but could once allow for in vitro studies on BBB permeability under pathophysiological conditions that are difficult to reproduce with current models (for review see [266]). Nevertheless, even simple, commercially available static systems can be very useful in small- to moderate-scale pre-clinical studies to facilitate early identification of slowly BBB penetrating compounds and/or substrates for the most important efflux transporters.

## Figures and Tables

**Table 1:** Range of reported TEERs and paracellular permeabilities of different in vitro BBB models.

Cell Type and Culture Conditions ^a^	TEER (Ω × cm^2^)	Paracellular Permeability ^b^		References
		*P_e_* (×10^−3^ cm/min)	*P_app_* (×10^−6^ cm/s)	
**Non-BBB cells**				
MDCK cells	72–300	-	1–3	[135,136,137]
Caco-2 cells	86–854	-	0.18–22.1	[137,138,139,140,141,142,143,144,145,146,147,148,149,150,151,152,153,154,155,156,157,158,159]
**Human hCMEC/D3 cells**				
Monoculture	2–100	1.3–1.6	9.3–22	[137,151,160,161,162,163,164,165,166,167,168,169,170,171]
Double co-culture	20–140	-	7	[165,167,172,173]
Triple co-culture	40	-	-	[174]
Dynamic co-culture	1000	-	-	[172]
Microfluidic system	30–1200	-	1.6	[151,164,175]
**Rat RBE4 cells**	-	-	-	
Monoculture	64–120	0.5–2.5	27–214	[137,176,177,178,179,180]
Double co-culture	200–510	0.2–1.5	-	[174,177,180]
Triple co-culture	300	0.2–1.5	-	[177]
**Mouse bEnd.3 cells**				
Monoculture	25–105	-	-	[181,182,183,184,185,186]
Double co-culture	20–130	-	16–23	[181,182,187,188]
Dynamic co-culture	250–300	-	-	[181,187]
Microfluidic system	1000	-	-	[189]
**Rodent primary cells**				
Monoculture	10–200	0.7	8–19	[151,190,191,192,193,194,195,196,197,198,199,200,201]
Double co-culture	20–780	0.3–0.4	2–9	[190,191,192,193,194,195,196,198,199,202,203,204,205,206,207]
Triple co-culture	170–400	0.03	0.8–6	[151,194,196,198,208,209,210,211]
Microfluidic system	600–1300	-	1.15	[151,192]
**Porcine primary cells**				
Monoculture	400–800	-	0.6–8	[212,213,214]
Double co-culture	700–1200	-	0.2–0.3	[191,212]
**Bovine primary cells**				
Monoculture	60–800	1.9	6-11	[215,216,217,218,219,220,221]
Double co-culture	130–2500	0.5–0.7	1–6	[215,218,220,222,223,224,225,226,227,228]
Triple co-culture	-	0.3–0.5	-	[229]

^a^ in order to facilitate comparison between the different culture conditions, articles using medium supplemented with hydrocortisone have not been included owing to its strong effect on TEERs/permeabilities; ^b^ permeabilities for commonly used paracellular permeability markers (sucrose, mannitol, sodium salt of fluorescein and/or Lucifer Yellow).

## Supplementary Materials

The following are available online at https://www.mdpi.com/article/10.3390/pharmaceutics13101542/s1: List of abbreviations, summary of important equations and parameters.

## Appendix A

**Table A1:** Results from studies comparing in vitro permeabilities in static co-culture models with PET-based in vivo data.

Permeability Measure		Compounds	Results	Ref.
In Vitro	In Vivo (PET)			
*PS_e_* in a non-contact co-culture model with human primary BMECs and astrocytes	*PS_in_* calculated from *K*_1_ in rats using the Renkin–Crone equation	11 fluoropyridinyl derivatives including [N′-aromatic and aliphatic]-thioureas, -ureas and -amides	Significant correlation between *PS_in_* and *PS_e_* (r^2^ = 0.985, *p* < 0.001), no correlation between *PS_in_* and logD or MW	[254]
*P_app(LA)_*/*P_app(AL)_* (=1/*ER*) in a non-contact co-culture model with human primary BMECs and astrocytes or in Caco-2 cells	*K*_1_/*k*_2_ in humans, where *k*_2_ is the rate constant for drug transfer from brain to arterial plasma	fluorodeoxyglucose, fluoro-L-DOPA, fluoro-A85380, befloxatone, flumazenil, raclopride	Significant correlation between *K*_1_/*k*_2_ and 1/*ER* in the double co-culture model (r^2^ = 0.90, *p* < 0.001), no correlation between *K*_1_/*k*_2_ and 1/*ER* in Caco-2 cells	[253]
*P_app_* in a non-contact co-culture model with human iPSC-derived BMECs and rat primary glial cells	*K*_1_ in humans determined in previous PET studies	loperamide, erlotinib, buprenorphine, fluoro-A85380, befloxatone, flumazenil, raclopride, verapamil	Significant correlation between *K*_1_ and *P_app_* (r^2^ = 0.83, *p* = 0.008), only major discrepancy observed for erlotinib (high *P_app_* vs. low *K*_1_)	[255]
