# Supplementary material for: Drug Penetration into the Central Nervous System: Pharmacokinetic Concepts and In Vitro Model Systems
Source: Pharmaceutics. 2021 Sep 23;13(10):1542. doi: 10.3390/pharmaceutics13101542 (PMC8538549; doi:10.3390/pharmaceutics13101542)
Supplement: Supplementary file 1 [file pharmaceutics-13-01542-s001.zip › pharmaceutics-1369806-supplementary.pdf]

pharmaceutics

MDPI

Supplementary Materials: Drug Penetration into the Central Nervous System: Pharmacokinetic Concepts and In Vitro Model Systems

Felix Neumaier, Boris D. Zlatopolskiy and Bernd Neumaier

1. List of abbreviations

ABC transporters - ATP-binding cassette transporters
AJs - adherens junctions
$A_{tot,brain}$ - total amount of a drug per g of brain tissue corrected for remaining drug in the vasculature
$A_{tot,brain+blood}$ - total amount of a drug per g of brain tissue including blood
$A_{tot,slice}$ - total amount of a drug per g of brain tissue in a brain slice
BBB - blood-brain barrier
BCSF - blood-cerebrospinal fluid barrier
BECF - brain extracellular fluid
bFGF - basic fibroblast growth factor
BICF - brain intracellular fluid
B_{max} - maximum concentration of binding sites for a ligand or tracer
BM - basement membrane
BMECs - brain microvascular endothelial cells
C_{BECF} - concentration of a drug in cerebral microdialysate (by definition unbound)
CBF - cerebral blood flow
C_{buffer} - concentration of a drug in buffer used for incubation of brain slices (by definition unbound)
C_{donor} - concentration of a drug in the donor compartment (to which the drug has been added) of a model BBB
$C_{donor(0)}$ - initial concentration of a drug in the donor compartment (to which the drug has been added) of a model BBB
$Cl_{bulkflow}$ - clearance of a drug from brain due to bulk flow of brain extracellular fluid into cerebrospinal fluid
Cl_{efflux} - efflux transporter clearance of a drug across the BBB out of the brain
Cl_{in} - net influx clearance of a drug into the brain (= sum of $Cl_{passive}$ and Cl_{uptake})
$Cl_{metabolism}$ - clearance of a drug from brain due to metabolism
Cl_{out} - net efflux clearance of a drug from the brain (= sum of $Cl_{passive}$, Cl_{efflux}, $Cl_{metabolism}$ and $Cl_{bulkflow}$)
clogD - logarithm of the calculated distribution coefficient of a drug between octan-1-ol and a buffer at pH 7.4
clogP - logarithm of the calculated partition coefficient of a drug between octan-1-ol and water
$Cl_{passive}$ - diffusional clearance of a drug across the BBB
Cl_{uptake} - active uptake transporter clearance of a drug across the BBB into the brain
CNS - central nervous system
$C_{receiver}$ - concentration of a drug in the receiver compartment (into which the drug is cleared) of a model BBB
CSF - cerebrospinal fluid
$C_{tot,brain}$ - total concentration of a drug in brain tissue at steady-state (for continuous infusion) or area under the drug concentration-time curve in brain tissue (for bolus injection)

$C_{tot,plasma}$ - total concentration of a drug in plasma at steady-state (for continuous infusion) or area under the drug concentration-time curve in plasma (for bolus injection)
$C_{tot,perf}$ - concentration of a drug in the perfusate used for *in situ* perfusion
ECM - extracellular matrix
ER - bidirectional efflux ratio for a drug across the endothelium of a model BBB
F - rate of perfusion (for calculation of PS_{in} from K_{in} determined by *in situ* perfusion) or rate of cerebral blood flow (for calculation of PS_{in} from K_{in} or K_1 determined by *in vivo* studies) [for details see 3.3 below]
$f_{u,plasma}$ - unbound fraction of a drug in plasma
$f_{u,brain}$ - unbound fraction of a drug in brain, determined *in vitro* with brain tissue homogenate [for details see 2.6 below]
$f_{u,dh}$ - unbound fraction of a drug in diluted brain tissue homogenate
GDNF - glia cell-derived neurotropic factor
HBD - number of hydrogen bond donor atoms of a drug
J - rate of appearance of a drug in the receiver compartment (into which the drug is cleared) of a model BBB
K_1 - net rate of drug transfer from blood to brain determined based on PET imaging + compartmental modelling
K_d - dissociation constant of a ligand or tracer for its binding sites
K_{in} - unidirectional transfer constant for the initial rate of brain entry by a drug, determined based ion *in vivo* studies [for details see 3.1 below] or *in situ* perfusion [for details see 3.2 below]
$K_{p,brain}$ - partition coefficient (ratio) of total brain to total plasma concentrations of a drug determined *in vivo* [for details see 2.1 below]
$K_{p,u,u,brain}$ - partition coefficient (ratio) of unbound brain to unbound plasma concentrations of a drug, usually determined based on a combination of *in vivo* and *in vitro* data [for details see 2.2 and 2.5 below]
logBB - logarithm of the partition coefficient $K_{p,brain}$ for a drug
logD - logarithm of the measured distribution coefficient of a drug between octan-1-ol and a buffer at pH 7.4
logP - logarithm of the measured partition coefficient of a drug between octan-1-ol and water
MPO - multi-parameter optimization
MRPs - multidrug resistance proteins
MW - molecular weight of a drug
P_{app} - apparent permeability coefficient for unidirectional transfer of a drug across a model BBB (=endothelium and cell-free membrane) [for details see 4.4 below]
$P_{app(AL)}$ - apparent permeability coefficient for unidirectional transfer of a drug across a model BBB (=endothelium and cell-free membrane) from abluminal to luminal compartment
$P_{app(LA)}$ - apparent permeability coefficient for unidirectional transfer of a drug across a model BBB (=endothelium and cell-free membrane) from luminal to abluminal compartment
P_e - permeability coefficient for unidirectional transfer of a drug across the endothelium of a model BBB [for details see 4.3 and 4.5 below]
$P_{e(AL)}$ - permeability coefficient for unidirectional transfer of a drug across the endothelium of a model BBB from abluminal to luminal compartment
$P_{e(LA)}$ - permeability coefficient for unidirectional transfer of a drug across the endothelium of a model BBB from luminal to abluminal compartment
P_m - permeability coefficient for unidirectional transfer of a drug across the cell-free membrane of a model BBB
PET - positron emission tomography
PGP/MDR1 - P-glycoprotein/multidrug resistance protein 1
pKa - ionization constant of the most basic center of a drug

PS_e - permeability surface area product for unidirectional transfer of a drug across the endothelium of a model BBB [for details see 4.2 below]
PS_{in} - permeability surface area product for transfer of a drug across the BBB into the brain (equivalent to Cl_{in}) [for details see 3.3 below]
PS_m - permeability surface area product for unidirectional transfer of a drug across the cell-free membrane of a model BBB
PS_t - total permeability surface area product for unidirectional transfer of a drug across a model BBB (=endothelium and cell-free membrane) [for details see 4.1 below]
S - exchange surface area of the endothelium in a model BBB or of the BBB *in vivo*
T - net perfusion time (for *in situ* perfusion)
$\mathbf{t_{1/2eq,in}}$ - intrinsic brain equilibrium half-time for a drug [for details see 3.4 below]
TEER - transendothelial electrical resistance
TGF-β - transforming growth factor β
TJs - tight junctions
TPSA - topological polar surface area of a drug
ΔV_{CL} - incremental clearance volume of a drug across a model BBB [for details see 4.1 below]
V_b - physiological volume of brain tissue
$\mathbf{V_{blood}}$ - volume of blood in the brain
$V_{receiver}$ - volume of the receiver compartment (into which the drug is cleared) of a model BBB
$\mathbf{V_T}$ - volume of distribution (=partition coefficient for total drug between brain and blood in PET terminology)
$V_{u,brain}$ - unbound volume of distribution of a drug in brain, determined *in vivo* by cerebral microdialysis [for details see 2.3 below] or *in vitro* with brain slices [for details see 2.4 below]

2. Equations related to the extent of brain penetration

2.1. Equation for determination of $K_{p,brain}$ based on in vivo studies

Background: To determine the distribution of a drug between blood and brain, multiple rodents receive a single bolus injection or continuous infusion of the drug of interest and the drug concentration in plasma and brain tissue homogenate is determined at different times after administration (for bolus injection) or after a steady-state has been reached (for continuous infusion).

$$K_{p,brain} = \frac{C_{tot,brain}}{C_{tot,plasma}}$$

$K_{p,brain}$ - partition coefficient (ratio) of total brain to total plasma concentrations of the drug
$C_{tot,brain}$ - total concentration of the drug in brain tissue at steady-state (for continuous infusion) or area under the drug concentration-time curve in brain tissue (for bolus injection)
$C_{tot,plasma}$ - total concentration of the drug in plasma at steady-state (for continuous infusion) or area under the drug concentration-time curve in plasma (for bolus injection)

2.2. Equation for determination of $K_{p,uu,brain}$ from $K_{p,brain}$ and $V_{u,brain}$

Background: To obtain a measure for the distribution of unbound drug, the partition coefficient of total brain to total plasma drug concentrations ($K_{p,brain}$, see 1.1) is corrected for drug binding to plasma proteins, for non-specific drug binding to brain tissue, and for the distribution of unbound drug in brain between extracellular and intracellular compartment.

$$K_{p,uu,brain} = \frac{K_{p,brain}}{f_{u,plasma} \times V_{u,brain}}$$

$K_{p,u,u,brain}$ - partition coefficient (ratio) of unbound brain to unbound plasma concentration of the drug

$K_{p,brain}$ - partition coefficient (ratio) of total brain to total plasma concentration of the drug
$f_{u,plasma}$ - unbound fraction of the drug in plasma
$V_{u,brain}$ - unbound volume of distribution of the drug in brain, determined *in vivo* by cerebral microdialysis (see 1.3) or *in vitro* with brain slices (see 1.4)

2.3. Equation for in vivo determination of $V_{u,brain}$ by microdialysis

Background: To obtain a measure for the fraction of unbound drug in brain and its distribution between extracellular and intracellular compartment, the unbound drug concentration in brain extracellular fluid after administration in rodents is determined by *in vivo* microdialysis and used (together with the total drug concentration in brain tissue determined post-mortem) to calculate the unbound volume of distribution of the drug in brain ($V_{u,brain}$).

$$V_{u,brain} = \frac{A_{tot,brain+blood} - V_{blood} \times C_{tot,plasma}}{C_{BECF}}$$

$V_{u,brain}$ - unbound volume of distribution of the drug in brain
$A_{tot,brain+blood}$ - total amount of the drug per g of brain tissue (including blood) determined post-mortem
V_{blood} - volume of blood in the brain
$C_{tot,plasma}$ - total concentration of the drug in plasma at steady-state (for continuous infusion) or area under the drug concentration-time curve in plasma (for bolus injection)
C_{BECF} - concentration of the drug in cerebral microdialysate (by definition unbound)

2.4. Equation for in vitro determination of $V_{u,brain}$ with brain slices

Background: To obtain a measure for the fraction of unbound drug in brain and its distribution between extracellular and intracellular compartment, a brain slice is incubated in buffer containing the drug and the (unbound) drug concentration in the buffer after the incubation is used (together with the total drug concentration in the brain slice) to calculate the unbound volume of distribution of the drug in brain ($V_{u,brain}$).

$$V_{u,brain} = \frac{A_{tot,slice}}{C_{buffer}}$$

$V_{u,brain}$ - unbound volume of distribution of the drug in brain (slices)
C_{buffer} - concentration of the drug in the incubation buffer (by definition unbound)
$A_{tot,slice}$ - total amount of the drug per g of brain slice tissue measured after the incubation

2.5. Equation for determination of $K_{p,uu,brain}$ from $K_{p,brain}$ and $f_{u,brain}$

Background: To obtain a measure for the distribution of unbound drug, the partition coefficient of total brain to total plasma drug concentrations ($K_{p,brain}$, see 1.1) is corrected for drug binding to plasma proteins and for drug binding to brain tissue.

$$K_{p,uu,brain} = K_{p,brain} \times \frac{f_{u,brain}}{f_{u,plasma}}$$

$K_{p,u,u,brain}$ - partition coefficient (ratio) of unbound brain to unbound plasma concentrations of the drug
$K_{p,brain}$ - partition coefficient (ratio) of total brain to total plasma concentrations of the drug
$f_{u,plasma}$ - unbound fraction of the drug in plasma
$f_{u,brain}$ - unbound fraction of the drug in brain, determined *in vitro* with brain tissue homogenate (see 1.6)

2.6. Equation for in vitro determination of $f_{u,brain}$ by equilibrium dialysis with brain homogenate

Background: To obtain a measure for the fraction of unbound drug in brain, brain tissue homogenate is diluted in buffer, equilibrated with the drug across a dialysis membrane and the unbound drug fraction in the diluted tissue homogenate is used (together with the dilution factor) to determine the unbound drug fraction ($f_{u,brain}$).

$$f_{u,brain} = \frac{1/D}{\left[\left(\frac{1}{f_{u,dh}}\right) - 1\right] + (1/D)}$$

$f_{u,brain}$ - unbound fraction of the drug in brain tissue homogenate
$f_{u,dh}$ - unbound fraction of the drug in diluted tissue homogenate
D - dilution factor

3. Equations related to the rate of brain penetration *in vivo* or *in situ*

3.1. Equation for determination of K_{in} based on in vivo studies

Background: To obtain a measure for the initial rate of brain entry by a drug, the concentration of the drug in brain at a given time after *in vivo* administration in rodents can be related to the amount of plasma exposure up to this point (i.e. the product of the area under the concentration-time curve in plasma and the unbound drug fraction in plasma).

$$K_{in} = \frac{A_{tot,brain}}{C_{tot,plasma} \times f_{u,plasma}}$$

K_{in} - unidirectional transfer constant for the initial rate of brain entry by the drug
$A_{tot,brain}$ - total amount of the drug per g of brain tissue (corrected for remaining drug in the vasculature)
$C_{tot,plasma}$ - total concentration of the drug in plasma at steady-state (for continuous infusion) or area under the drug concentration-time curve in plasma (for bolus injection)
$f_{u,plasma}$ - unbound fraction of the drug in plasma

3.2. Equation for determination of K_{in} based on in situ perfusion

Background: To obtain a measure for the initial rate of brain entry by a drug, the concentration of the drug in brain at a given time after *in situ* perfusion with drug-containing buffer can be related to the amount of drug exposure up to this point (i.e. the product of the concentration of drug in the perfusate and the net perfusion time).

$$K_{in} = \frac{A_{tot,brain}}{C_{tot,perf} \times T}$$

K_{in} - unidirectional transfer constant for the initial rate of brain entry by the drug
$A_{tot,brain}$ - total amount of the drug per g of brain tissue (corrected for remaining drug in the vasculature)
$C_{tot,perf}$ - concentration of the drug in the perfusate
T - net perfusion time

3.3. Equation for determination of PS_{in} from K_{in}

Background: To obtain the permeability surface area product (PS_{in}) for transfer of a drug across the BBB from the unidirectional transfer constant K_{in}, the Renkin–Crone equation is used to account for the rate of drug delivery by cerebral blood flow (for K_{in} determined by *in vivo* studies) or the rate of drug delivery by the perfusate (for K_{in} determined by *in situ* perfusion) respectively. The same equation can be used to calculate PS_{in} from the kinetic PET parameter K_1.

$$PS_{in} = -F \times ln\left(1 - \frac{K_{in}}{F}\right) = -F \times ln\left(1 - \frac{K_1}{F}\right)$$

PS_{in} - permeability surface area product for transfer of the drug across the BBB into the brain

K_{in} - unidirectional transfer constant for the initial rate of brain entry by the drug, determined by *in vivo* studies (see 2.1) or *in situ* perfusion (see 2.2)
F - rate of perfusion (for K_{in} determined by *in situ* perfusion) or rate of cerebral blood flow (for K_{in} or K_1 determined by *in vivo* studies)
K_1 - net rate of drug transfer from blood to brain (determined by PET imaging + compartmental modelling)

3.4. Equation for determination of $t_{1/2eq,in}$ from PS_{in} and $f_{u,brain}$

Background: To obtain a measure for the time required for drug equilibration between blood and brain that takes into account the effects of non-specific drug binding to brain tissue, PS_{in} and $f_{u,brain}$ can be used to calculate the intrinsic brain equilibrium half-time.

$$t_{1/2eq,in} = \frac{ln2 \times V_b}{PS_{in} \times f_{u,brain}}$$

$t_{1/2eq,in}$ - intrinsic brain equilibrium half-time
V_b - physiological volume of brain tissue
PS_{in} - permeability surface area product for transfer of the drug across the BBB into the brain
$f_{u,brain}$ - unbound fraction of the drug in brain, determined *in vitro* with brain tissue homogenate (see 1.6)

4. Equations related to the rate of BBB penetration in *in vitro* models

4.1. Equation for determination of PS_t for transfer of a drug across a model BBB

Background: To determine the permeability surface area product for transfer of a drug across a model BBB separating two compartments, the drug is added to one of the compartments (=donor compartment), its concentration in both compartments (=donor and receiver compartment) is repeatedly measured over time and used to calculate the incremental clearance volume ΔV_{CL} (using the equation below) for each time point. ΔV_{CL} is then plotted as a function of time and the slope of the linear part of the resulting line corresponds to the total permeability surface area product for unidirectional transfer of the drug across the model system (PS_t).

$$\Delta V_{CL} = \frac{C_{receiver} \times V_{receiver}}{C_{donor}}$$

ΔV_{CL} - incremental clearance volume
C_{donor} - drug concentration in the donor compartment (to which the drug has been added)
$C_{receiver}$ - drug concentration in the receiver compartment (into which the drug is cleared)
$V_{receiver}$ - volume of the receiver compartment (into which the drug is cleared)
PS_t - total permeability surface area product for unidirectional transfer of the drug across the model system (i.e. endothelium and cell-free membrane)

4.2. Equation for determination of PS_e from PS_t and PS_m

Background: To obtain an estimate of the permeability surface area product for unidirectional transfer of a drug across the endothelium forming the model BBB (PS_e), PS_t can be corrected for the drugs permeability surface area product for transfer across the cell-free membrane on which the endothelium is grown (PS_m).

$$\frac{1}{PS_e} = \frac{1}{PS_t} - \frac{1}{PS_m}$$

PS_t - total permeability surface area product for unidirectional transfer of the drug across the model system (i.e. endothelium and cell-free membrane)
PS_e - permeability surface area product for unidirectional transfer of the drug across the endothelium
PS_m - permeability surface area product for unidirectional transfer of the drug across the cell-free membrane

4.3. Equation for determination of P_e from PS_e

Background: To obtain an estimate of the permeability coefficient for unidirectional transfer of a drug across a modell BBB (which is independent of the surface area of the endothelium forming the model BBB), the corresponding permeability surface area product (PS_e) and the known exchange surface area of the endothelium (S) can be used to calculate the permeability coefficient for unidirectional transfer of the drug across the endothelium.

$$P_e = \frac{PS_e}{S}$$

PS_e - permeability surface area product for unidirectional transfer of the drug across the endothelium
P_e - permeability coefficient for unidirectional transfer of the drug across the endothelium
S - exchange surface area of the endothelium

4.4. Equation for determination of P_{app} for transfer of a drug across a model BBB

Background: To determine an apparent permeability coefficient for unidirectional transfer of a drug across a model BBB separating two compartments (P_{app}), the drug is added to one of the compartments (=donor compartment) and its concentration in the other compartment (=receiver compartment) is repeatedly measured over time.

$$P_{app} = \frac{J}{C_{donor(o)} \times S}$$

J - rate of appearance of the drug in the receiver compartment (into which the drug is cleared)
$C_{donor(0)}$ - drug concentration in the donor compartment (to which the drug is added) at the start of the experiment
P_{app} - apparent permeability coefficient for unidirectional transfer of the drug across the model system (i.e. endothelium and cell-free membrane)

4.5. Equation for determination of P_e from P_{app}

Background: To determine the true permeability coefficient for unidirectional transfer of a drug across a model BBB (P_e) from the corresponding apparent permeability coefficient (P_{app}), the latter can be corrected for the permeability coefficient for unidirectional transfer of the drug across the cell-free membrane on which the endothelium is grown (P_m).

$$\frac{1}{P_{app}} = \frac{1}{P_e} - \frac{1}{P_m}$$

P_{app} – apparent permeability coefficient for unidirectional transfer of the drug across the model system (i.e. endothelium and cell-free membrane)
P_e - permeability coefficient for unidirectional transfer of the drug across the endothelium
P_m - permeability coefficient for unidirectional transfer of the drug across the cell-free membrane

4.5. Equation for determination of the bidirectional ER from (apparent) permeability coefficients

Background: The permeability coefficients for unidirectional transfer of a drug into both directions across a model BBB can be used to calculate the bidirectional efflux ratio (ER).

$$ER = \frac{P_{e(AL)}}{P_{e(LA)}} = \frac{P_{app(AL)}}{P_{app(LA)}}$$

ER - bidirectional efflux ratio for the drug across the endothelium
$P_{e(AL)}$ - permeability coefficient for unidirectional transfer of the drug across the endothelium from abluminal to luminal compartment
$P_{e(LA)}$ - permeability coefficient for unidirectional transfer of the drug across the endothelium from luminal to abluminal compartment

$P_{app(AL)}$ - apparent permeability coefficient for unidirectional transfer of the drug across the model system (i.e. endothelium and cell-free membrane) from abluminal to luminal compartment
$P_{app(LA)}$ - apparent permeability coefficient for unidirectional transfer of the drug across the model system (i.e. endothelium and cell-free membrane) from luminal to abluminal compartment
